# Postoperative pain and antibacterial effect of 980 nm diode laser versus conventional endodontic treatment in necrotic teeth with chronic periapical lesions: A randomized control trial

**DOI:** 10.12688/f1000research.16794.1

**Published:** 2018-11-15

**Authors:** Dina A. Morsy, Maged Negm, Alaa Diab, Geraldine Ahmed

**Affiliations:** 1 Department of Endodontics, Faculty of Oral and Dental Medicine, Cairo University, Cairo, 11553, Egypt

**Keywords:** Diode laser, intracanal irradiation, necrotic pulp, periapical lesion, endodontic

## Abstract

**Background: **Many challenges encounter the endodontist, especially when dealing with necrotic teeth with chronic periapical lesions. Postoperative pain may be induced following conventional endodontic therapy and total eradication of the bacteria is almost unachievable even with recently available techniques. In recent years, diode laser usage in the endodontic field has gained acceptance. Thus, this study aimed to investigate the ability of the diode laser (DL) to decrease postoperative pain and achieve root canal sterility.

**Methods: **56 patients with anterior teeth with chronic periapical lesions in upper anterior teeth were randomly divided into two groups (n = 28). All patients were treated with two visits of conventional root canal treatment with ProTaper Universal. The DL group: root canals were irradiated with 200 µm fiber optic at both visits; the control group (Endo): the DL fiber was placed in root canal with no activation. Bacterial samples were collected from all the cases at each step of the treatment. Pain levels were evaluated using a numerical rating scale preoperatively, and after 6, 12, 24, 48 hours and 7 days. Bacterial count was used to detect both aerobic and anaerobic bacterial load.

**Results: **The qualitative pain scores revealed statistically significant lower pain levels in the DL group compared with the Endo group at all time intervals (P<0.001), except preoperatively where there was no significant difference. There was a statistically significant lower bacterial count for both aerobic and anaerobic bacteria in the DL group compared with the Endo group in both S3 samples (after laser application) and S4 samples (bacterial colonization) (P<0.001).

**Conclusion**: The 980 nm diode laser may be a successful adjunct to conventional endodontic treatment of necrotic cases with chronic periapical lesions in terms of postoperative pain and root canal disinfection.

**Trail registration:** PACTR201511001275414 (date: 23 ^rd^ September 2015)

## Introduction

The therapeutic goal of endodontic treatment in cases of necrotic teeth with chronic periapical lesions is the creation of a sterile, bacteria-free environment in the tooth and at the apex, including the periodontal tissue and the surrounding apical bone ^1^. There are two main complicating factors preventing achievement of this goal: the complicated anatomical root configuration and the special characteristics of the resident bacterial flora, which makes it sometimes inaccessible even with recent available armamentarium ^2^. In recent years, intracanal laser irradiation has been used in root canal preparation ^3^, gaining acceptance for its disinfection ability as adjunct to the conventional mechanical instrumentation and irrigation protocols available ^4, 5^. It was also reported that the use of laser therapy may result in decreased postoperative pain ^6^. Recently, the diode laser (DL) 980 nm was introduced and its use in root canal disinfection was established ^4^.

### Objectives

This study aimed to evaluate the ability of the 980 nm DL to reduce the post-operative pain and intracanal bacteria when compared with conventional endodontic treatment.

## Methods

### Trial design

This study is a parallel randomized controlled trial, with an allocation ratio of 1:1 and a superiority framework.

This study was approved by the Research Ethics Committee of the Faculty of Oral and Dental Medicine, Cairo University (15-9-19).

This article was written in concordance with the CONSORT checklist 2010 ( Supplementary File 1).

### Participants

Patients received in the outpatient clinic of the Endodontic Department, Faculty of Dentistry, Cairo University in the duration between March 2016 and March 2017 were invited to participate. In total 56 participants were included in the study after fulfilling the inclusion criteria and after signing an informed consent.

*Inclusion criteria:* Adult patients with average age between 18 and 35 years; medically free patients; patients suffering from necrotic pulp in maxillary central incisors permanent teeth with: closed apex, associated with or without sinus tract, radiographic evidence of periapical radiolucency; patients with healthy dental and periodontal status; positive patients’ acceptance for participation in the study.

*Exclusion criteria:* Illiterate patients; pregnant woman (as the hormonal changes may alter the pain perception); patients having systemic disorder; teeth that have: open apex, extra coronal restorations, greater than grade I mobility, pocket depth greater than 4 mm, non restorable tooth, previous endodontic treatment; patients taking analgesics 12 hours before the intervention; patients who had received antibiotics in the last month; patients with acute pain at the time of intervention.

## Sample size

To assess DL versus conventional endodontic regarding the postoperative pain, an independent t test was done. It was estimated that a total of 50 patients would be required for the detection of a difference between groups using a two-tailed α of 0.05 and a power of 0.80 if the absolute difference in periapical lesions is 0.37 mm with SD 0.46 as reported in Markovick *et al*. in 2006. To compensate losses during the follow-up this number should be increased to 56 patients (10% more than the calculated). Sample size was calculated using G* Power program (2).

### Interventions

All patients were treated by a single endodontic over two visits after signing an informed consent.

***First visit.*** At the first visit, all patients recorded their pain level preoperatively using a numerical rating scale (NRS; see below for details) *.* The teeth were locally anaesthetized (Articaine in 4% solution with epinephrine in concentration of 1:100000 (Ultracaine- dental forte, Germany)

Before isolation, antisepsis of the oral cavity was performed by rinsing for 1 min with 10 mL chlorhexidine gluconate mouth wash 0.2 %. The teeth were properly isolated with rubber dam ^1^. An access cavity was performed. Patency of the root canal was obtained using stainless steel hand k- files size #15 (MANI- MANI, INC. Industrial Park, Utsunomiya, Tochigi, Japan). The root canals were irrigated with 1 ml sterile saline solution. The first microbial samples (S1) were collected to assess the initial colonizers of the root canals using 3 sterile paper points which were inserted into the root canal for 1 min each with pumping movements. They were immediately placed inside sterile tubes containing a reduced transport medium of thioglycolate. Working length was determined using an electronic apex locator (DENTA PORT ZX (J.Morita, Irvine, Japan)), then confirmed with intraoral periapical radiograph. Mechanical preparation was performed with the ProTaper Universal Ni Ti system up to #F4 file for all the cases. In total 10 ml of 2.5% sodium hypochlorite was used for irrigation between each file and the next using a 25-gauge needle. 5 ml of 17% EDTA (Calix E, DHARMA research, Miami, USA) was used at the end of the procedure to remove the smear layer. 5 ml of saline solution was the final irrigant used to neutralize all the previously used solutions. The second microbial samples (S2) representing the antibacterial effect of the mechanical preparation were obtained with the same procedure as the (S1) samples.

According to the randomization and sequence generation, the patients were allocated into two groups (n = 28/group). 

**Figure 1.**
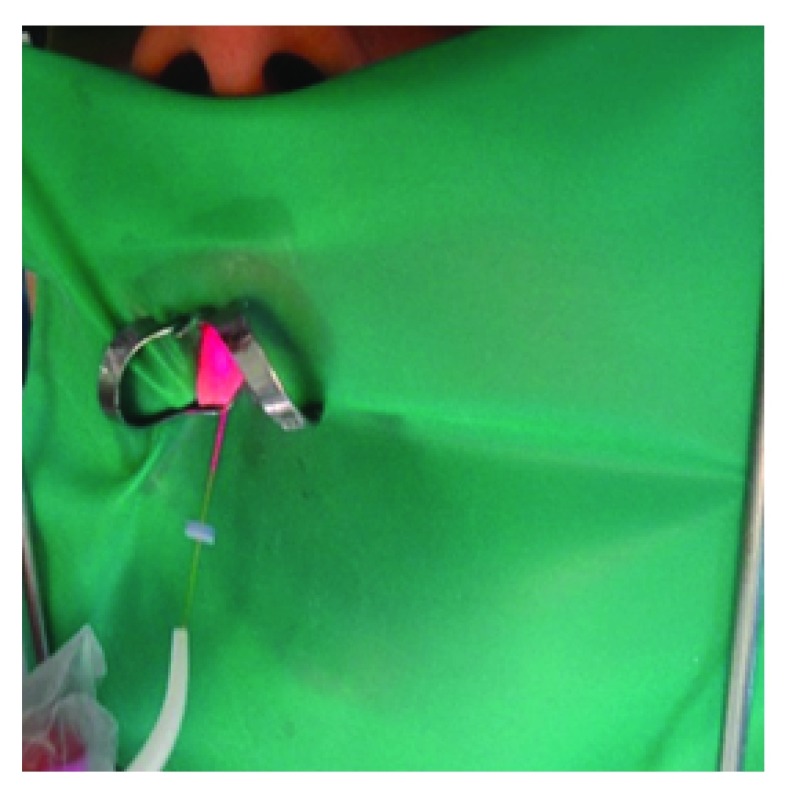
Intracanal application of diode laser.

*Experimental (DL) group*: Root canals were irradiated with 980 nm diode laser coupled with optical fiber 200 µm (Lite medics, Italy) with setting 1.2-watt power, in pulsed mode. The irradiation protocol was a 5 sec irradiation followed by a 10 sec pause, which constituted one lasing cycle. The lasing cycle was performed four times for each tooth. The tip was positioned 1 mm short of the apex. This was followed by activation during which it was slowly dragged at a speed of approximately 2 mm/ sec in a way that the root canals were irradiated from the apical to the coronal portion, in a helicoidal movement touching the canal walls. This was done to ensure equal diffusion of light inside the root canal lumen ( Figure 1). The third microbial samples (S3) were collected as mentioned before to evaluate the effect of DL on the bacterial count.

*Control (Endo) group:* Conventional endodontic treatment as above, after which the fiber optic was placed inside the root canals without activation (placebo) with no bacterial sample collected at this stage.

At the end of the first visit a piece of sterile cotton was placed in the pulp chamber and all teeth was dressed with intermediate restorative material (IRM) as a temporary filling (Dentsply, Latin America)

***Second visit.*** At the second visit, one week later; the canals of both groups were accessed under rubber dam. Canals were irrigated with 1 ml sterile saline solution. The fourth microbial samples (S4) were taken to assess the recolonization of the bacteria and were collected from both groups. The same procedures of the first visit, the intracanal irradiation for the DL group and placebo for the Endo group was performed. The fifth microbial samples (S5) were collected to assess the status of root canal just before the obturation.

Each root canal was obturated with the modified single cone technique using the ProTaper Universal gutta-percha points size F4 and gutta-percha points size 25 (META, Biomed, Republic of Korea) as auxiliaries with ADSEAL resin-based root canal sealer (META BIOMED CO., LTD. Chungbuuk, Korea). All the teeth were restored with IRM as a temporary filling. At the end of the second visit, all patients were instructed to record pain level on the pain scale chart after 6, 12, 24 and 48 hours and after 7 days. The patients were instructed to submit the pain scale charts after the 7 ^th^ day. Patients were referred for final restorations. Any patient who reported the intake of an analgesic during this period was excluded from the study.

### Primary outcome: Pain evaluation

The NRS consisted of a line anchored by two extremes "No pain" and "the worst pain". The patients were asked to mark the chart at the point that represented their level of pain from 0 to 10.

Pain level was assigned to one of 4 categorical scores: No pain (0), Mild (1–3), Moderate (4–6) and Severe (7–10).

### Secondary outcome: Microbiological analysis

The bacterial count method was used ^2^. Once the samples arrived to the microbiology department, Cairo University, the tubes containing the thioglycolate (transport medium) (Thioglycollate broth U.S.P alternative, Oxoid microbiology product, LTD, England) with the paper points were placed in microcentrifuge and vortexed for 30 sec. 100 µl aliquots of the vortexed samples were placed in a new sterile tube containing 1 ml of thioglycolate to obtain 1/10 concentration to assess the microbial load of common aerobes and anaerobes found in each root canal. However, no attempt was made to identify the specific microbial flora during the process. The effect of the treatment in each group, the mechanical preparation (S2), the Diode laser irradiation (S3) in the DL group only, the bacterial recolonization (S4) and the bacterial count just before obturation (S5), were compared.

*Aerobic bacterial culture:* 50 µl of these diluted samples were transferred to BHI agar plates (Oxoid microbiology product, LTD, England) and cultured under aseptic conditions, followed by incubation at 37 ^o^ C for 24 hours for the aerobic bacteria. The number of bacterial colonies in each plate was counted and reported as colony forming units per milliliter (CFU/ml).

*Anaerobic bacterial culture:* The other 50 µl of these diluted samples were transferred to BHI agar plates under aseptic conditions, the agar plates were placed in an anaerobic sealed jar with Gas-Pak (Gas-Pak system) (Oxoid microbiology product, LTD, Basingstoke, Hants, England) and anaerobic indicator (Anaerobic indicator, BR0055B.Oxoid Ltd. Basingstoke, Hants, England) were incubated for 48 hours at 37 ^o^ C. Eventually, the number of bacterial colonies in each plate was counted and reported as CFU/ml.

### Blinding and randomization

Double blinding was implemented in this study by the assessor and the statistician concerning evaluation of the post-operative pain intensity and microbiological evaluation. However, single blinding was implemented only by the statistician concerning the results of periapical lesion size. The blinded assessors were asked to fill a chart for each outcome with the number corresponding to each patient without knowing which group the participants were related to.

A random sequence was generated by computer software ( http://www.random.org/) in the Center of Evidence Based Dentistry, Cairo University. The table was kept with the assistant supervisor. Four-folded numbered papers were packed in opaque sealed envelopes to be chosen by the patients after entering the study. The opaque envelopes contained the numbers of each random sequence to assign the patient to either the experimental (DL) or control group (Endo).

The assistant supervisor assigned the participants to the experimental or control groups according to the randomization table. After confirmation on patient eligibility with the assistant, the operator applied the treatment procedure assigned to that patient. 

### Statistical analysis

Statistical analysis was performed using SPSS 19 (SPSS, Chicago, IL, USA). As data related to patients’ age and bacterial colony formation were parametric, significance of the difference between both groups was evaluated using unpaired t test. Chi square test was used to compare the qualitative pain scores. The level of significance was set at P<0.05.

## Results

In total, 56 patients were included in the study. A CONSORT flow diagram can be seen in Supplementary File 2.

Demographic data, age and gender, had no significant difference between the two groups (P=0.1967 and 0.053, respectively; Table 1 and Table 2).

**Table 1.** Age of participants.

Age (years)	Diode laser group	Control group
**Mean**	**25.28 ^a^**	**26.25 ^a^**
**SD**	**5.11**	**5.47**
**Min**	**18**	**18**
**Max**	**35**	**35**
**t-test**	**1.31**	
**P-value**	**0.1967 ^ns^**	

SD= standard deviation, Min= minimum, Max= Maximum, t=unpaired t test, *: p<0.05 (Significant), ns: P> 0.05 (not significant)

**Table 2.** Gender distribution of participants.

Gender	Diode laser group	Control group
**Male**	14	10
**Female**	14	18
**X ^2^**	3.733	
**P-value**	0.053 ^ns^	

X ^2^: Chi square test, Significance level: P<0.05, ns: non-significant. SD= standard deviation, Min= minimum, Max= Maximum, t=unpaired t test, *: p<0.05 (Significant), ns: P> 0.05 (not significant)

**Figure 2.**
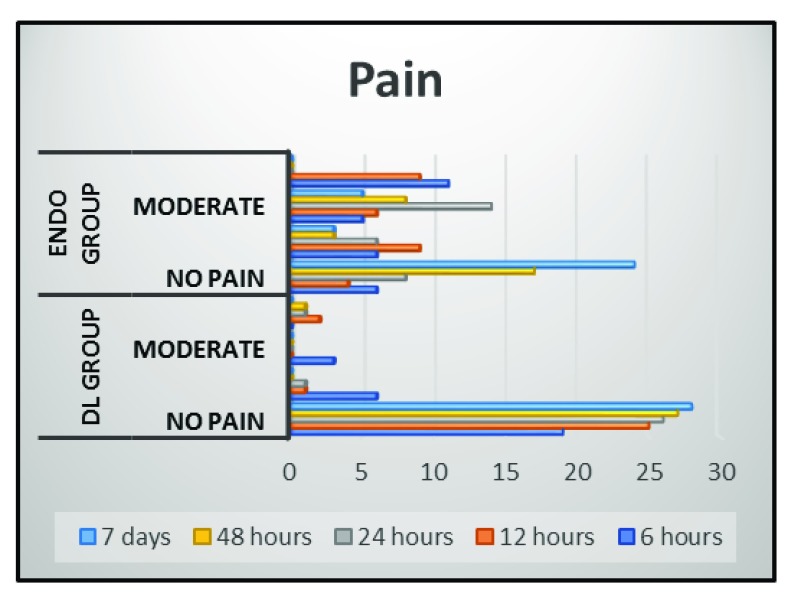
Column chart showing qualitative score of pain in patients enrolled in both groups. Means with different small letters in the same column indicate statistically significance difference, means with different capital letters in the same row indicate statistically significance difference. *; significant (p<0.05), ns; non-significant (p>0.05).

**Figure 3.**
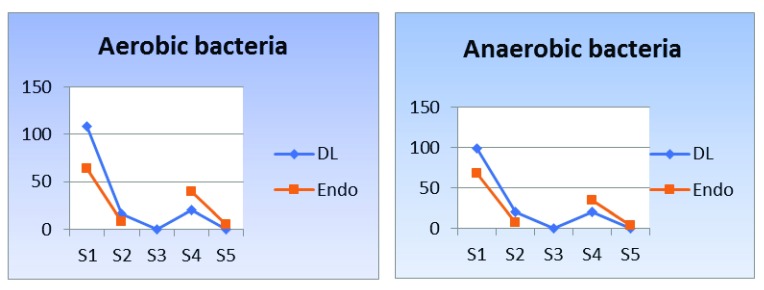
Line chart showing bacterial count ( **a**) aerobic and ( **b**) anaerobic. Means with different small letters in the same column indicate statistically significance difference, means with different capital letters in the same row indicate statistically significance difference. *; significant (p<0.05), ns; non-significant (p>0.05).

The qualitative pain scores revealed statistically significant lower pain levels in the DL group compared with the Endo group at 6, 12 and 24 hours (P<0.001), and at 48 hours and 7 days (P=0.002 and 0.044, respectively), while preoperatively there was no statistical significance difference (P=1.0) ( Figure 2 and Table 3). The results of the bacterial count of both aerobic and the anaerobic bacteria of the DL group showed statistically significant reduction in the bacterial count than the Endo group at S4 and S5 (Aerobic: P< 0.01 and 0.002, respectively; anaerobic: P< 0.002 and 0.012, respectively; Figure 3 and Table 4 and Table 5).

**Table 3.** Mean and SD values of pain intensity of different time periods within each group.

	Pain		Pre-operative	6 hrs	12hrs	24hrs	48hrs	7 days	X ^2^	P-value
**Diode laser** **group**	**No pain**	**n**	**28**	**19**	**25**	**26**	**28**	**28**	**33.25**	<0.001 *
		**%**	**100**	**67.9**	**89.3**	**92.9**	**100**	**100**		
	**Mild**	**n**	**0**	**6**	**1**	**1**	**0**	**0**		
		**%**	**0**	**21.4**	**3.6**	**3.6**				
	**Mod**	**n**	**0**	**3**	**0**	**1**	**0**	**0**		
		**%**	**0**	**10.7**		**3.6**				
	**Severe**	**n**	**0**	**0**	**2**	**0**	**0**	**0**		
		**%**	**0**		**7.2**					
**Control group**	**No pain**	**n**	**28**	**6**	**4**	**8**	**17**	**24**	**65.93**	<0.001 *
		**%**	**100**	**21.4**	**14.3**	**28.6**	**60.7**	**85.7**		
	**Mild**	**n**	**0**	**6**	**9**	**6**	**3**	**3**		
		**%**	**0**	**21.4**	**32.1**	**21.4**	**10.7**	**10.7**		
	**Mod**	**n**	**0**	**5**	**6**	**14**	**8**	**1**		
		**%**	**0**	**17.9**	**21.4**	**50**	**28.6**	**3.6**		
	**Severe**	**n**	**0**	**11**	**9**	**0**	**0**	**0**		
		**%**	**0**	**39.4**	**32.1**					
**X ^2^ test**	**X ^2^**		**3.744**	**18.26**	**34**	**28.101**	**14.273**	**8.077**		
	**P-value**		**1**	<0.001 *	<0.001 *	<0.001 *	0.002 *	0.044 *		

DL: Diode laser group, ES: endosurgery group, hrs: hours, n: number of patients, %: percentage of patients, mod: moderate pain, SD, standard deviation. X ^2^: Chi square test, significance level: P<0.05, *significant

**Table 4.** Mean and standard deviation values of the bacterial count (CFU/ml ×10 ^4^) of aerobic bacteria of different groups.

Variables	Aerobic bacteria					
	**Diode laser group**		**Control group**		**P-value**	
	Mean	SD	Mean	SD		
**S1**	108.21 ^aA^	36.05	63.50 ^aB^	18.77	0.012*	
**S2**	16.00 ^cA^	4.42	8.29 ^cB^	2.99	0.006*	<0.001*
**S3**	0.286 ^dC^	0.09	-	-		
**S4**	13.678 ^bB^	10.77	40.25 ^bA^	38.45	0.01*	
**S5**	0.00 ^dB^	0.00	5.25 ^dA^	1.04	0.002*	
**P-value**	< 0.001*		< 0.001*			

**Table 5.** Mean and standard deviation values of the bacterial count (CFU/ml ×10 ^4^) of anaerobic bacteria of different groups.

Variables	Anaerobic bacteria					
	Diode laser group		Control group		P-value	
	Mean	SD	Mean	SD		
**S1**	98.79 ^aA^	31.37	67.50 ^aB^	16.12	0.047*	
**S2**	19.750 ^cA^	6.79	7.32 ^cB^	2.32	0.016*	<0.001*
**S3**	0.036 ^dC^	0.009	-	-	-	
**S4**	8.75 ^bB^	7.428	36.607 ^bA^	11.68	0.002*	
**S5**	0.00 ^dB^	0.00	3.61 ^dA^	1.09	0.012*	
**P-value**	< 0.001 *		< 0.001 *			

Full de-identified data for each participant, including demographic data, pain scores at all time intervals, and bacterial count of both aerobic and anaerobic bacteria Click here for additional data file. Copyright: © 2018 Morsy DA et al. 2018 Data associated with the article are available under the terms of the Creative Commons Zero "No rights reserved" data waiver (CC0 1.0 Public domain dedication).

## Discussion

This study aimed to evaluate the ability of the 980nm diode laser to decrease postoperative pain and disinfect the root canal and to evaluate whether the diode laser could be a successful adjunctive aid to conventional endodontic treatment.

Over time, various laser types have been developed and are used in different dentistry fields ^7^. Among the various lasers, diode lasers are the most frequently used ^8^. The active medium of the 980 nm diode laser is a solid-state semiconductor made of indium, gallium and arsenide. Diode lasers have several advantages: extreme compactness, affordability, ease of operation, simple setting-up, versatility and small size ^8^. Diode wavelengths are highly absorbed in hemoglobin and melanin and have little absorption in dental hard tissue. They are also highly absorbed by water ^9^, which provides the laser with the advantage of acting selectively and precisely ^8^.

In the present study, the intracanal irradiation was done using the pulsed mode to decrease the risk of thermal damage on external root surface and thus decrease the postoperative pain and favor healing of periapical area ^10^. The temperature on the root canal walls rapidly decreases as the intracanal irradiation with the activated 200 µm fiber-optic is directed from apical to coronal direction rapidly. Thus, guaranteeing that the surrounding tissue is only marginally affected and damage of periodontal tissues or the underlying bone should not be expected ^9^.

The 980 nm diode laser use an optical flexible fiber 200 µm to deliver the beam to the target area, probably distributing homogenously the light inside the root canal for a more efficient photoreaction ^7^. Garcez *et al*. ^11^ achieved higher antimicrobial effect when they used the optical fiber in disinfection of the root canal. Diode lasers have demonstrated excellent clinical benefits ^12^.

In this study, the NRS scale was used to record postoperative pain. Jamison *et al*. ^13^, reported that, the NRS has greater sensitivity to change in pain intensity. Amelia and Barbara ^14^ reported that the NRS represents interval levels so it can provide data for parametric analysis.

The results of this study showed that the DL group had statistically significant lower pain levels than the Endo group at all tested time intervals (6, 12, 24, 48 hours and 7 days). These results are in accordance with the findings of Berk *et al*. ^15^ and Pawar *et al*. ^16^, who reported that the use of diode laser in root canal irradiation showed significantly lower pain at 8, 24, 48 hours and 7 days postoperatively when compared to conventional treatment. Tuner *et al*. ^17^, found that, usually after conventional RCT of chronic cases, which is the situation in this trial, the case become acute when the process of healing starts, thus patients are at risk of experiencing postoperative pain which was not the situation in the DL group. The exact mechanism by which the use of laser results in decreasing post-operative pain is still unknown. Some authors proposed some mechanisms by which the diode laser relieves pain: Pawar *et al*. ^16^ and Bjordal *et al*. ^18^ found that the diode laser acts on chronic pain and has an anti-inflammatory effect by decreasing PGE2, bradykinin, histamine, acetyl choline and serotonin, also the diode laser was proved to decrease the production of substance P. Our results concerning postoperative pain in the cases of chronic apical periodontitis, when treated with diode laser, showed promising results.

It is generally accepted that the development of periapical diseases are pathologic features of polymicrobial bacterial infection and its components which stimulate bone resorption ^19^. Thus, in this study the effect of the treatment on both the aerobic and the anaerobic bacterial count were assessed following the same methodology of Garcez *et al*. ^20^ and Bonsor *et al*. ^2^. This technique was selected due to its ability to detect exclusively the viable bacteria and also the correlation proved previously, by many studies ^21, 22^, between negative cultures at time of obturation and more favorable treatment outcomes.

In this study, the antibacterial results showed statistically significant lower bacterial count in the S3 samples of DL group than the S2 samples of both groups. It also showed significantly lower bacterial recolonization in S4 samples of the DL group than the Endo group. The S5 samples of the DL group resulted in significantly lower bacterial count than the Endo group, which may favor the treatment outcomes ^21, 22^. Our findings are in accordance with the findings of Garcez *et al*. ^11^ and Gutknecht *et al*. ^4^, who found that the use of diode laser resulted in significant decrease of the intracanal bacterial load. The high antibacterial effect of diode laser may be explained by the fact that the near infrared lasers are absorbed to small extent by dentin. This is important for the efficient disinfection as the laser is not absorbed by the superficial dentin but rather penetrates deep into the intertubular dentin ^1^. Vaarkamp *et al*. ^23^ and Odor *et al* ^. 24^ provided an explanation for this way of light propagation, as they described the ability of enamel prisms and dentinal tubules to act as an optical fiber and thus allowing the diode laser to be more effective in deep layers of dentin. According to Gutknecht *et al*. in 2008 ^9^, the ND: YAG, 810 nm diode laser and 980 nm diode laser are the only wavelengths that showed high transmission through hydroxyapatite and water. Thus, it can be used successfully for the disinfection of root canals.

Diode laser radiation has a bactericidal effect by altering the bacterial cell wall. Microbiologists ^25^ talk about a permanent destruction of the cell membrane, which is commonly in correlation with direct heat having an impact on the bacteria. The diode laser exerts a photo-thermal effect on the bacteria ^26^. It also, exerts a photo-disruptive effect on the unreachable bacteria ^26^.

 Guteknecht *et al*. ^4^ demonstrated that diode laser light can penetrate up to >1000 μm into the dentin. Thus, it can be an effective means for disinfection of the root canal system together with conventional biomechanical instrumentation reaching areas, which were considered earlier non-reachable.

### Strengths and limitations

This study is a randomized clinical trial conducted on a relatively big sample size patients, in real clinical settings and was conducted efficiently. It proposes an alternative way for treatment of necrotic teeth with chronic periapical lesions efficiently and without postoperative pain.

The following limitations should be considered: this study didn’t evaluate if there is a difference between single visit or two visit approach when the intracanal diode laser is used on postoperative pain and bacterial count of necrotic teeth with chronic periapical lesions.

Further *in vivo* and immunological studies are needed to identify the exact mechanism by which the intracanal Diode laser resulted in decreasing postoperative pain.

## Conclusions

Intracanal diode laser irradiation has the ability to decrease the postoperative pain experienced after conventional root canal treatment in cases of necrotic teeth with periapical lesions. Implementation of suitable wavelengths, together with conventional methods of cleaning and shaping, can effectively sterilize the root canals, dentin and periapical area and decrease the bacterial recolonization. Thus, based on the findings of this study, it may be concluded that the 980 nm diode laser can be used as an adjunct to conventional endodontic therapy. 

## Data availability

The data referenced by this article are under copyright with the following copyright statement: Copyright: © 2018 Morsy DA et al.

Data associated with the article are available under the terms of the Creative Commons Zero "No rights reserved" data waiver (CC0 1.0 Public domain dedication). 

F1000Research: Dataset 1. Full de-identified data for each participant, including demographic data, pain scores at all time intervals, and bacterial count of both aerobic and anaerobic bacteria. , https://doi.org/10.5256/f1000research.16794.d224351 ^27^

## Supplementary material

Supplementary File 1: Completed CONSORT checklist.

Supplementary File 2: CONSORT flow diagram.

Supplementary File 2: Full study protocol.
