# Supplementary material for: Postoperative pain and antibacterial effect of 980 nm diode laser versus conventional endodontic treatment in necrotic teeth with chronic periapical lesions: A randomized control trial
Source: F1000Res. 2018 Nov 15;7:1795. [Version 1] doi: 10.12688/f1000research.16794.1 (PMC6659763; doi:10.12688/f1000research.16794.1)
Supplement: Supplementary file 3 [file f1000research-7-18360-s0002.tgz › ba04a8d2-0e51-4672-b3be-41c100f78628_CONSORT_flow_diagram_of_experimental_stages_and_group_distribution.docx]

**CONSORT flow diagram of experimental stages and group distribution**

**Patient selection**

**Enrollment** n= 56

**Inclusion criteria:**

1. Adults patients.
2. Medically free patients.
3. Patients with necrotic pulp with chronic periapical lesions in mature maxillary anterior incisors
4. Healthy periodontal status

**Exclusion criteria**

1. i. Pregnant females
2. ii. Medically compromised
3. iii. Teeth that have: open apex, greater than grade I mobility, pocket depth greater than 4 mm, non restorable.
4. Iv- Acute pain at the time of intervention.

**Eligibility assessment**

**Included**

**n=56**

**Excluded**

**n=19**

**Preoperative x-ray**

**Randomization**

**n=56**

**First visit**

1^st^ microbiological sample (S1)

**Endodontic treatment:**

Access cavity, patency and saline irrigation

Mechanical preparation till F4 ProTaper

Irrigation NaOCl and 17% EDTA

2^nd^ microbiological sample (S2)

**DL group**

**Allocation**

**n=56**

**Endo group**

- Intracanal Diode laser application

n=28

- Temporization
- Placebo n=28
- Temporization

3^rd^ microbiological sample (S3)

**Second visit**

4^th^ microbiological sample (S$)

(Canal recolonization)

3^rd^ microbiological sample (S4)

(Canal recolonization)

**Access to the root canal**

**And saline irrigation**

5^th^ microbiological sample (S5)

(just before obturation)

4^th^ microbiological sample (S5)

(just before obturation)

Mechanical preparation

Mechanical preparation and Diode laser irradiation

Post-operative pain assessment (n=28)

Post-operative pain assessment (n=28)

**Obturation and restoration**
