# Supplementary material for: Postoperative pain and antibacterial effect of 980 nm diode laser versus conventional endodontic treatment in necrotic teeth with chronic periapical lesions: A randomized control trial
Source: F1000Res. 2018 Nov 15;7:1795. [Version 1] doi: 10.12688/f1000research.16794.1 (PMC6659763; doi:10.12688/f1000research.16794.1)
Supplement: Supplementary file 4 [file f1000research-7-18360-s0003.tgz › febb4876-3b07-4959-8cc7-c075534c7a56_Protocol.docx]

**qPOSTOPERATIVE PAIN, ANTIBACTERIAL EFFECT AND HEALING OF CHRONIC PERIAPICAL LESIONS TREATED WITH DIODE LASER VERSUS ENDODONTIC SURGERY: A Randomized Control Trial**

**Protocol**

Submitted to the Faculty of Oral and Dental Medicine-Cairo University in partial fulfillment of the requirement for the Protocol for Doctorate Degree in Endodontic

BY

DINA AHMED ALI MORSY

BDS, MSc (Cairo University)

Faculty of Oral and Dental Medicine, Cairo University

(2015)

- **Roles and responsibilities:**

**Investigator**:***:* Dina Ahmed Ali Morsy**

Doctor degree student

Department of Endodontic

Faculty of Oral and Dental Medicine

Cairo University

**Chief Supervisor**: ***Chief Supervisor:***

***Dr.*Maged Mohamed Negm Dr. Alaa Diab**

Professor Professor

Department of Endodontic Department of Endodontic

Faculty of Oral and Dental Medicine Faculty of Oral and Dental Medicine

Cairo University Cairo University

- **Funding:** Self-funding.
- **Trial Registration:**To be later online registered on PACTR website

**Introduction**

Many challenges encounterthe endodontist specially when dealing with necrotic teeth with periapical lesion and bony defect. This may lead the endodontist to shift to the endodontic surgery in many cases. Especially with the complicated anatomical root configuration which make it almost impossible to achieve 100 % bacterial reduction and thus promote healing and decrease postoperative pain.As thetherapeutic goal of each root canal treatment is creation of a sterile, bacteria-free environment both in the tooth and at the apex, including the periodontal tissue and the surrounding apical bone. At this time osteoblasts would be able to complete the healing process in the apical area ^(1)^. There are two factors that complicate achieving sterility in the tooth: the complicated anatomical root configuration and the special characteristics of the resident bacterial flora which make it sometimes impossible to reach the residual apical bacteria^(2).^

A flare-up following all endodontic procedures occurs in 1.4-16% of the patients ^(3)^. Other clinical studies reported that postoperative pain occurs in 25-40% of all the endodontic patients ^(4)^. There are few studies on postoperative pain following the surgical root canal treatment ^(5-7)^. According to Chon and Pitt Ford, 90% of their patients experienced pain between the first three to five hours, 82% after 24 hours and 72% after 48 hours^(8)^. Flare-up is upsetting to both patient and dentist. The patient has either pain or swelling or a combination of both a few hours to a few days after endosurgery procedure ^(9)^. Flare-ups are more likely to occur in necrotic cases than in vital cases ^(10)^. In case of non vital pulp, the root canals are usually infected, especially in the presence of chronic periapical lesions. Patients who had received nonsurgical followed by surgical endodontic treatment were approximately four times more likely to develop chronic pain after successful endodontic treatment ^(11)^. Effective control of intracanal microbial load before obturation is a key element that leads to a high success rate of root canal treatment ^(12-14)^.

Nowadays the use of low level laser therapy has been widely accepted to eliminate residual bacteria within the root canal system ^(15&16)^, reduce periapical inflammation and pain ^(17)^. It was found that low level laser therapy reduce the postoperative recovery period of up to a third of the time described in the literature^(18)^. For all of the above mentioned reasons many in-vivo ^(19, )^ and in-vitro studies are done to prove the efficacy of use of diode laser with the conventional cleaning and shaping procedures to promote healing of chronic periapical lesion ( up to 5 mm) thus limiting the need of the endodontic surgery to selective cases.

The purpose of this study is aimed to answer a clinical question whether the use of 980 nm diode laser result in f healing of the periapical lesion and reduction of bacteria from root canal system and decrease the need of endodontic surgery.

# Trial Design:

Type: Parallel randomized controlled trial

Allocation ratio: 1:1

Framework: Superiority

# Aim of the study:

# The aim of this study is to investigate the use of diode laser inside the root canals of necrotic teeth with chronic periapical lesion and its antibacterial effect, healing and its ability to decrease the postoperative pain without the need of endodontic surgery.

PICO approach:

P: Patients with anterior teeth with periapical lesions.

I: Endodontic treatment+ intra canal diode laser application

C: Apicectomy (surgical root canal treatment).

O:

|  | **Outcome measure** | **Tool for measurement** | **Unit of measurement** |
| --- | --- | --- | --- |
| Primary | Pain | Numeric rating scale (NRS)^(22)^ | Ordinal |
| Secondary | Healing of periapical lesion  (Bone density) | Cone beam computed tomography | Hounsfield unit (HU) |
|  | Intracanal bacterial flora  (Ex-vivo study) | Bacterial culture  ^(23)^ | Colony forming unit/ mm of blood agar medium |

S→ Randomized control study

**Justification of the used NRS and not VAS:**

1. The visual analog scale (VAS) is the most difficult scale to use in the clinical practice to record the pain intensity and with the highest failure rate ^(24)^.
2. The NRS is interval level so it can provide data for parametric analysis, the scale is easy to administer and record. ^(24)^.
3. The NRS has greater sensitivity to change in the pain intensity ^(25)^.

# Research question:

- Will the use of diode laser as an adjunct method make difference in incidence of postoperative pain, healing and reduction of bacteria in case of teeth with periapical lesion when compared to conventional method following endodontic surgery and will it restrict the endosurgery procedure to specified cases?

**Materials and methods**

- **Subjects and Methods:**

**1. Setting and location:**

*** Source of patients**: Out patients of the clinic of endodontic at the faculty of oral and dental medicine, Cairo University, Urban area, Cairo governorate.

*** Operator Qualification:** Post Graduate Master’s Degree Student.

*** Dental units**: Adec 200 U.S.A.

* **X-ray machine**: ViVi, S.r.I, Italy.

* **X-ray film**: Intraoral periapical Kodac Dental film, speed D, size 2.

**Laser device:** Diode laser (980nm), Lite Medics, Italy

1. **Eligibility criteria :**
2. *Inclusion criteria:*
3. Adults
4. Medically free patients in order not to affect the healing of the wound
5. Upper anterior teeth with necrotic pulp and chronic periapical lesion with evidence of radiological periapical lesion (<5mm)
6. Healthy dental and periodontal status
7. *Exclusion Criteria*:
8. Teeth with open apex
9. Pregnant or nursing female patients
10. Acute pain at time of the intervention

- **Final Diagnosis:**
- Maxillary asymptomatic anterior teeth.
- On using electric pulp tester it gives response of non vital pulp
- On radiographic examination it shows a periapical lesion
- **Intervention:**

All patients treated in this study will have to fill a full medical and dental history using a schematic dental chart. Each tooth will be evaluated for vitality (sensitivity) of pulp tissues using electric pulp tester

The patients will be randomly divided into 2 groups:

- ***Experimental group***: diode laser application inside the root canal after complete cleaning and shaping of the root canal
- ***Control group:*** conventional endodontic surgery (Apicectomy)
- **Sequence of Procedural steps:-**

1. **The experimental group ( Root Canal Treatment)**
2. Patients presented to the endodontic department with asymptomatic anterior teeth with chronic periapical lesion will be selected.
3. Periapical radiograph will be taken for the selected cases to determine the presence of apical lesion
4. The patient who will fulfill the inclusion criteria will be send to the center cone beam computed tomography for initial imaging to detect the initial bone density and the size of the lesion (<5mm)
5. In the experimental group, tooth will be anaesethized using the infiltration technique by local anesthesia Articaine in 4% solution with epinephrine in conc 1:100000^[[1]](#footnote-1)^
6. Access cavity will be performed using round bur and fine tapered stone.
7. Rubber dam will be applied to the treated tooth to ensure proper isolation.
8. The surrounding area and the pulp chamber will be irrigated with 5 ml of Chlorohexidine solution 2% to ensure that the crown of tooth will be with minimal microbial load
9. Canal will be accessed using k-file # 15^[[2]](#footnote-2)^, approximately to the apical portion of the canal. File will be moved back and forth to remove necrotic tissues. Then root canal will be irrigated with 1 ml sterile saline solution and canal will be dried with 3 sterile paper points which will be left inside the root canal for 1 min each.
10. After which all three points will be placed inside a fresh sterile bottle with a sterile nutrient broth ( 1^st^ microbiological sample)
11. Mechanical preparation will be done using the crown down technique while working 1 mm short to the working length. The rotary ProTaper file system will be used^[[3]](#footnote-3)^ in the endodontic motor^[[4]](#footnote-4)^ (X-smart) with the adjusted torque and speed according to the manufacturer’s instructions. The rotary files will be introduced inside the treated root canal with EDTA gel^[[5]](#footnote-5)^ProTaper File F_4_ will be the average preparation diameter of the apical portion which will be enough to allow the optical fiber to reach the full working length
12. Alternating use of 10 ml of NaOCl 2.5% and 3% H_2_O_2_as irrigation among each file using a 27 gauge needle
13. 5 ml of 17% EDTA will be used at the end of the procedure
14. 5 ml of sterile saline will be the final irrigant to neutralize all the previously used irrigants
15. The canals will be dried using another 3 paper points each left for 1 min and will be stored as before ( 2^nd^ microbilogical sample)
16. The canals will be irradiated with diode laser (980 nm) coupled with the optical fiber 2oo µm ^[[6]](#footnote-6)^
17. Then another 3 paper points will be inserted in the canal ( 3^rd^ microbiological sample)
18. At the end of the first visit Ca (OH)_2_^[[7]](#footnote-7)^, will be placed in the canal. A piece of cotton will be placed in the pulp chamber and tooth will be dressed with a temporary restorative material IRM^[[8]](#footnote-8)^
19. One week later a 2^nd^ endodontic visit will be held with exactly the same procedure as visit one. The 1^st^ microbiological sample of the 2^nd^ visit will be to assess the recolonization of the bacteria.
20. Then each root canal will be obturated using the lateral condensation technique with TopSeal^[[9]](#footnote-9)^
21. Tooth will be restored with composite as the final restoration (Filtek Z350) ^[[10]](#footnote-10)^
22. **The control group (endodontic surgery)**
23. Local anaesthesia administration as in the experimental group
24. In the first and second visit conventional endodontic treatment as in the experimental group except for the laser irradiation.
25. In the day of surgery, local anaesthesia infiltration
26. Full thickness mucoperiosteal flap with a sulcular incision and vestibular relieving incisionwill be raised to expose the buccal plate
27. Root tips will be exposed with round. 3 mm root resection will be done using a fissure bur
28. Curettage of the lesion with excavators. To control the bleeding, Racestyptine^[[11]](#footnote-11)^ will be applied to the bony cavity using a cotton pellet
29. Retrograde cavity preparation using a diamond coated ultrasonic tip ^[[12]](#footnote-12)^
30. MTA ^[[13]](#footnote-13)^will be used as the material for root end filling. A pear shaped bur will be used to burnish the filling
31. Surgical flap repositioning and suture with 3O silk suture.
32. Post surgical indication: 7 days of Augmentine 1000 mg (twice per day) and Brufen 400 mg ( 3 times per day)
33. Suture removal after one week.
34. **Light source**

The 200 µm fiber optic will be places inside the root canal, 1 mm from the working length or till resistance is felt ^(26)^. A spiral movement from the apical to crown touching the canal walls to ensure equal diffusion of light inside the root canal lumen. According to the literature ^(27,28)^, four irradiation of 5 sec each will be performed. Each irradiation has a rest period of 5 sec to avoid the temperature increase above the threshold of 7º C.

1. **Microbiological analysis**

Once the samples arrive to the microbiology department, Cairo University, the paper points will be removed from the transport medium and placed in 1.5 ml microcentrifuge with BHI broth and positioned in a vortex for 30 sec. 100 µl aliquots will be added to wells of a 96 well plate for serial dilution. Diluted samples will be placed on blood agar plates. At each stage of the treatment (initial, after endodontic procedure and after laser irradiation in the first group)

The CFU_s_ will be counted ^(29).^For this procedure two assessors will be first calibrated and then the inter-rater (examiner) reliability will be confirmed. For the calibration, the assessors (specialized microbiologists from the microbiology department, Kasr-Einy, Cairo University) will be asked to provide readings of already assessed blood agar plates (not related to the study samples) representing different records of the colony forming units (CFU). These calibrated and blinded examiners will then analyze the radiographs of the study sample.

1. **Pain evaluation**

Pain will be evaluated using the Numerical Rating Scale (NRS). Patients will be instructed to fill in the scale at preoperative, 6, 24 and 48 hours and 7 days postoperative. Any patient in the experimental group will be excluded if he takes analgesics after either the first or the second visit.

1. **Bone density evaluation**

- The cone beam computed tomography will be used to assess the changes in bone density with three records for each patient preoperative, after 3 months and after 6 months. In the pretreatment CBCT images, the lowest bone density units were recorded from scans with a 2.25-mm2 area in regions where the periapical lesions were localized in HUs, without differentiating between cysts and granulomas. The final measurements will be done by using the same parameters. For this procedure two assessors will be first calibrated and then the inter-rater (examiner) reliability will be confirmed. For the calibration, the assessors (specialized radiologists from the dental radiology department, CBCT center, Kasr-Einy, Cairo University) will be asked to provide readings of already assessed images (not related to the study samples)representing different bone densities. These calibrated and blinded examiners will then analyze the radiographs of the study sample.^(30)^
- **Inter-Rater (examiner) reliability**
- The inter-rater reliability is done to determine that the two assessors will be consistent in their readings with almost no disagreement in their separate readings.
- The % of agreement between the raters will be calculated to determine the inter-rater reliability (according to William M., 2006, types of reliability).
- Inter-rater reliability test (kappa coefficient) will be performed to assess the interpreters’ agreement. As described by Landis and Koch ^(31)^: 0.00-0.20, slight agreement; 0.21-0.40, fair agreement; 0.41-0.60, moderate agreement; 0.61-0.80, substantial agreement and 0.81-1.00 almost perfect agreements.
- **Outcomes:**
- **Primary Outcome (pain):**

The NRS scale is an 11 point scale (0-10 scale) where the end points are the extremes of "no pain" and "worst pain". Patients will be asked to choose the mark that represents their level of pain from 0 to 10, Pain level will be assigned as follow:

# 0 reading represents “no pain”

# 3 readings represent “mild pain”

# 4- 6 readings represent “moderate pain”

# 7- 10 readings represent “severe pain”

- **Secondary outcome (healing of periapical lesion):**

The cone beam computed tomography will be used to assess the changes in bone density with three records for each patient preoperative, after 3 months and after 6 months using the Hounsfield unit (HU).

- **Secondary outcome (antibacterial effect)**

The microbial load will be evaluated at each stage of the treatment (initial, after endodontic procedure and after laser irradiation in the first group) in both first and second visit. The CFU_s_ will be counted.

- **Participant timeline:**

1. Patients will be selected from the clinic of endodontic after eligibility.
2. They will be randomized and pretreatment diagnosed. A periapical radiograph will be done to confirm the presence of a periapical lesion
3. They will go to the CBCT center to confirm that the lesion size is less than 5 mm and to record the initial bone density.
4. Patients will be treated in two visits for the experimental group. For the control group an extra visit to perform the endosurgery will be held.
5. They will record postoperative degree of pain at 6, 12, 24, 48 hours and 7 days after the second visit for the experimental group and after the surgery in the control group.
6. Patients will be reminded to record the pain by phone then patients will retain pain dairy to the operator.
7. They will go to the CBCT center to assess the healing of the periapical lesion at 3 and 6 months postoperative.
8. Follow up for each group according to the following periods:

| **periods** | **Recruitment** | **Diagnosis** | **Periapical radiograph** | **CBCT imaging** | **Randomization** | **Follow up for postoperative pain** | **Follow up for healing** |
| --- | --- | --- | --- | --- | --- | --- | --- |
| Zero |  |  |  |  |  |  |  |
| 6 h |  |  |  |  |  |  | - |
| 12 h |  |  |  |  |  |  | - |
| 24 h |  |  |  |  |  |  | - |
| 48 h |  |  |  |  |  |  | - |
| 7 days |  |  |  |  |  |  | - |
| 3 months |  |  |  |  |  | - |  |
| 6 months |  |  |  |  |  | - |  |

- **Sample Size:**

To assess Diode Laser Versus Endodontic Surgery regarding Healing Of Chronic Periapical Lesions, an independent t test will be done. It was estimated that a total of 50 patients would be required for the detection of a difference between groups using a two-tailed α of 0.05 and a power of 0.80 if the absolute difference in periapical lesions is 0.37 mm with SD 0.46 as reported in Markovick et al .,2006. To compensate losses during the follow-up this number should be increased to 56 patients (10% more than the calculated)

Sample size was calculated using G*Power program (University of Düsseldorf, Düsseldorf, Germany).

# Recruitment:

Patients are recruited for our clinical trial from the clinic of endodontic at the faculty of oral and dental medicine, Cairo University to meet the target sample size.

- **Randomization:**

1. **Sequence Generation :**

A random sequence will be generated by computer software,

(http://www.random.org/).

The table will be kept with the assistant supervisor.

1. **Allocation Concealment Mechanism :**

- Eightfolded numbered papers will be packed in opaque sealed envelopes to be dragged by thepatients.
- Operator will open the envelope and use the instrumentation technique assigned to that patient. The opaque envelopes will contain the numbers of each random sequence for both groups (root canal treatment and irradiation with diode laser) or (surgical root canal treatment).

# Implementation:

The assistant supervisor or co-investigator is the one who will generate the random sequence, assign the participants to the intervention or control groups and the only one who knows whether Aor B represents the intervention or the control group.

**Blinding:**

- The study will be double- blinded which is participants and the assessor.
- Participants will not know which group they will be treated with after they will choose the folded paper inside the opaque envelopes which contain the numbers of each random sequence for both groups.
- Assessor who will assess all resultsdata will not know which group the participants related to (fig. 9-11)
- **Data collection, management, and analysis**
- Baseline Data collection:
- Baseline data will be collected by the operator through a report (Fig.1-5), which comprises of 2 charts (one concerning general health and one concerning the chief complaint related events).
- The report will be anonymous where patients identified by their serial numbers (the first letters of the first and last name and date of birth) only will be registered.
- The full detailed personal data of the patient will be written in a separate sheet having the patient's serial number for further contact with patient, this sheet can be only seen by the operator and the assistant supervisor.
- Outcome data collection: (Fig 6-7)

Primary outcomes will be collected by the operator through (NRS) which is scale consisting of numbers from 0 to 10:

# 0 reading represents “no pain”

# 3 readings represent “mild pain”

# 4- 6 readings represent “moderate pain”

# 7- 10 readings represent “severe pain”

- Secondary outcomes will be collected by the operator from the microbiological department and the CBCT center
- **Statistical analysis:**

Data will be analyzed using IBM SPSS advanced statistics (Statistical Package for Social Sciences), version 21 (SPSS Inc., Chicago, IL). The score of pain will be described as median and range or interquartile range, as appropriate.The analyses of the pain score will be done by Mann-Whitney U test to test the significance between the 2 groups.The periapical lesions and bacterial colony will be described as mean and SD or median and range as appropriate, comparison between 2 groups will be done by independent t test or Mann Whitney as appropriate at the end of follow up. A p-value less than or equal to 0.05 will be considered statistically significant. All tests will be two tailed.

G*Power program (University of Düsseldorf, Düsseldorf, Germany).Faul, F., Erdfelder, E., Lang, A.-G., & Buchner, A. (2007). G*Power 3: A flexible statistical power analysis program for the social, behavioral, and biomedical sciences. Behavior Research Methods, 39, 175-191.

- **Data monitoring:**

There will not be a Data Monitoring Committee for the following reasons:

- Interim analysis is not involved in the trial so periodic inspection of the accumulating outcome data is not required.
- Short duration of the trial.
- Minimal risks of the trial.
- **Harms**

Adverse events are minimal or rare. However, if any harm is seen in the participants either in intervention or control groups they will be recorded and reported at the end of the trial. It will be documented as part of routine monitoring not as outcome. The treatment will be according to the harm:

- Pain: administration of Analgesics.
- Swelling: hot fomentation, mouth rinse with salty worm water, antibiotic will be administrated.
- Allergic reaction: referral for a physician for corticosteroid therapy.

# Auditing

In this trial, auditing will be performed in order to preserve the integrity of the trial; the audit will review the processes of participant enrolment, consent, eligibility, allocation to study groups and adherence to trial interventions and policies to protect participants.

- **Ethics and dissemination**
- **Research ethics approval:**
- This protocol and the template informed consent form will be reviewed by the Ethics Committee of Scientific Research, faculty of oral and dental medicine, Cairo University.
- Each of the protocol, informed consent forms in Arabic and English language (fig. 11 and 12), recruitment materials and any modifications will be done later on also will be reviewed and approved by the ethical review Committee.
- **Protocol amendments:**

Any administrative changes or modifications of the protocol or modification of registered online protocol will be notified by the ethical committees.

# Consent:

- The trial will be discussed by the research with patients.
- Patients will then able to informed discussion with the researcher.
- Researcher will obtain written consent from patients willing to participate in the trail.
- All consent form will be in Arabic language.
- **­Confidentiality:**
- The confidentiality of the patients can be obtained through usage of password protected system to secure all local databases
- In this system a coded file will be used to identify all patients' personal data such as name, age address, phone number, work address.
- The safe secure storage of all files will be done through storage in locked cabinets with limited access.
- **Declaration of interests:**

No conflict of interests known.

# Access to data:

The validity of results from our interventional trial can be verified only by the Project Principal Investigators, trial supervisor and trial assistant supervisor, who will have direct full access to their own site’s complete final data sets.

# Ancillary and post-trial care:

Post-trial care planned, patient will be referred to fixed prosthodontics specialist for crown placement after completion of the root canal treatment.

# Dissemination policy:

We are intending to disseminate the results of the trial in the library of the faculty, in the Egyptian dental journal and in the journal of endodontic.

# Informed consent materials:

Patient informed consent:

Ethics Committee will revise and modify according to their guidelines.

Consent: (Fig 11 & 12).

- **References:**

1. Asnaashari M and Asnaashari N. Clinical Application of 810nm Diode Laser and Low Level Laser Therapy for Treating an Endodontic Problem A Case Presentation. Journal of Lasers in Medical Sciences 2011; 2( 2) : 82-85
2. Moritz A. Oral Laser Application. Quintessenz Verlags- GmbH, Berlin. 2006
3. Sipaviciute E. and Maneliene R. Pain and flare up after endodontic treatment procedures. Baltic dental and maxillofacial journal, 2014, 16: 25-30.
4. Holstein A., Hargreaves K. and Neidman R. evaluation of NSAIDS for treating post endodontic pain. Endodontic topics, 2002, 3:3-13.
5. Seymour R. , Meechan J., and Blair G. postoperative pain after apicectomy. A clinical investigation. Int endod j, 1986, 19: 242-247.
6. Kvist Tand Reit C. Postoperative discomfort with surgical and nonsurgical endodontic retreatment. Endod dent traumatol, 200, 16: 71-74.
7. Tseis I, Fuss Z, Lin S., Tilinger G and Peled M. Analysis of postoperative symptoms following surgical endodontic treatment. Quintessence Int, 2003, 34:756-760.
8. Gracia B., Larrazabal C., Penarrocha Ma and Penarrocha Mi. postoperative pain after root end resection and filling. Med Oral patol Oral Cir Buc, 2008, 13(11): 726-729.
9. Walton R, Fouad A. Endodontic Interappointment Flare-ups: a prospective study of incidence and related factors. J Endod 1992;18: 172-177.
10. Seltzer S, Naidorf IJ. Flare-ups in endodontics: I. Etiological factors. J Endod 1985;11:472-478.
11. Polycarpou N., Ng Y., Canavan D., Moles D. and Gulbivala k. Prevalence of persistent pain after endodontic treatment and factors affecting its occurrence in cases with complete radiographic healing. International endodontic journal, 2005, 3: 196-173.
12. Peters LB, van Winkelhoff AJ, Buijs JF, Wesselink PR. Effects of instrumentation, irrigation and dressing with calcium hydroxide on infection in pulpless teeth with periapical bone lesions. Int Endod J 2002; 35: 13–21.
13. Peciuliene V, Maneliene R, Balcikonyte E, Drukteinis S, Rutkunas V. Microorganisms in root canal infections: a review. Stomatologija 2008;10:4–9.
14. Siqueira Jr and Rocas IN. Clinical implications and microbiology of bacterial persistence after treatment procedures. J Endod 2008;34:1291–301
15. Kreisler M., kohnan W., Beck M., Gotz H., Jansen B and d’Hoedt B. Efficacy of NaOCl/ H_2_O_2_ irrogation on GaAIAs laser in decontamination of root canal in vitro. Surg Med, 2003, 32(3) 189-196.
16. Romeo U., Palaia G., Pacific L., Ripari F., Gambarini G., Moroni C. Tarsitani G. and Petti. Antimicrobial activity of Nd:YAG laser in endodontics. Journal of Dental Research, 2003
17. Asnaashari M., Mohebi S. and Paymonpour P. Pain reduction using low level laser irradiation in single visit endodontic treatment. Journal of lasers in medical sciences, 2011, 4(2): 139-143.
18. Simunovic Z. and Simmunovic K. lasers in dentistry. Lasers in medicine. 2000: 477-492.
19. Garcez A., Nunez S., Hamblin M. and Ribeiro M. Antimicrobial effects of photodynamic therapy on patients with necrotic pulp and periapical lesions. Journal of endodontics, 2008, 34(2): 138-142.
20. Gutknecht N., Franzen R., Schippers M. and Lampert F. Bactericidal effect of a 980 nm diode laser in root canal wall dentine of bovine teeth. Journal of clinical laser medicine and surgery, 2004, 1(22): 9-13.
21. Agron B., Aliu X., Mucaj A. and Hysenaj N. The efficacy of diode laser Nd:YAG in root canal and periapical lesions. European scientific journal, 2014, 10 (36) : 61-70.
22. Payer M., Jakse N. , Pertl C., Truschnegg A., Lechner E and Eskici A. the clinical effect of LLLT in endodontic surgery:A prospective study on 72 cases. 2005, 3(100):375-379.
23. Jurič IB, Plečko V, Pandurić DG and Anić I. The antimicrobial effectiveness of photodynamic therapy used as an addition to the conventional endodontic re-treatment: a clinical study. Photodiagnosis Photodyn Ther, 2014, 11(4): 549-555.
24. Amelia W. and Barbara H. Pain : A review of three commonly used pain rating scales. Journal of clinical nursing, 2005, 14: 789- 804.
25. Jamison R., Gracely R., Raymond S., Levine J., Marina B., Herman T., Daly M., Fram D and katz N.Comparative study of electronic versus paper VAS ratings: A randomized, crossover trial using healthy volunteers. Pain, 2002, 99: 341-347.
26. Romeo U., Palia G., Koenblit R., Tenore G and Del Vecchio A. Endodontic treatment of periapical chronic periodontitis A Laser Assisted Technique, 2010.
27. Gutknecht N, Franzen R, Meister J, Vanweersch L, Mazian M: Temperature evolution on human teeth root surface after diode laser assisted endodontic treatment. Lasers in Medical Science 2005;20:99–103
28. Bergmans L, Moisiadis P, Teughels W, Van Meerbeek B, Quirynen M, Lambrechts P: Bactericidal effect of Nd:YAG laser irradiation on some endodontic pathogens ex vivo. Intern. Endod. Journal 2006,39:547–557
29. Garcez A., Neto J., Sellera D., Fregnani E. Effetc of antimicrobial photodynamic therapy and surgical endodontic treatment on bacterial load reduction and periapical lesion healing. 3 years follow up. Accepted mnuscipt 1-6-2015:1-15.
30. Kaya S., YavizI., Uysal I. and Akkus Z. Measuring bone densiy in healing periapical lesion by using cone beam computed tomography: A clinical investigation. Journal of endodontics, 2012, 1(38): 28-31
31. Landis J, Koch G. The measurement of observer agreement for categorical data. Biometrics. 1977;33(1):159–174

Fig 1

**Appendix**

Fig 2

Fig 3

Fig 4

fig.5

Fig 6

***Numerical Rating Scale (1 _10)***

**اسم المريض: ................................... اسم الطبيب: ........................................**

**رقم المريض: ( ) رقم تليفون الطبيب: ( )**

يرجي وضع علامة علي المقياس ادناة لاظهار شدة ألمك.

1. **Patient training (١) تدريب**

| 0 | 1 | 2 | 3 | 4 | 5 | 6 | 7 | 8 | 9 | 10 |
| --- | --- | --- | --- | --- | --- | --- | --- | --- | --- | --- |

الم لا يحتمل لا يوجد ألم

1. **Preoperative (٢) قبل العلاج**

| 0 | 1 | 2 | 3 | 4 | 5 | 6 | 7 | 8 | 9 | 10 |
| --- | --- | --- | --- | --- | --- | --- | --- | --- | --- | --- |

الم لا يحتمل لا يوجد ألم

1. **After 6 hours (٣) بعد سته (6) ساعات**

| 0 | 1 | 2 | 3 | 4 | 5 | 6 | 7 | 8 | 9 | 10 |
| --- | --- | --- | --- | --- | --- | --- | --- | --- | --- | --- |

الم لا يحتمل لا يوجد ألم

Fig. 6

**(5) After 12 hours (٤) بعد اثنا عشر (١٢) ساعة**

| 0 | 1 | 2 | 3 | 4 | 5 | 6 | 7 | 8 | 9 | 10 |
| --- | --- | --- | --- | --- | --- | --- | --- | --- | --- | --- |

1. الم لا يحتمل لا يوجد ألم

**(6) After 24 hours (٥) بعد أربع و عشرون (٢٤) ساعة**

| 0 | 1 | 2 | 3 | 4 | 5 | 6 | 7 | 8 | 9 | 10 |
| --- | --- | --- | --- | --- | --- | --- | --- | --- | --- | --- |

1. الم لا يحتمل لا يوجد ألم

**(٦) بعد ثمان وأربعون (٤٨) ساعة (7) After 48 hours**

| 0 | 1 | 2 | 3 | 4 | 5 | 6 | 7 | 8 | 9 | 10 |
| --- | --- | --- | --- | --- | --- | --- | --- | --- | --- | --- |

الم لا يحتمل لا يوجد ألم

**(7) بعد سبعة أيام (7) After 7 days**

| 0 | 1 | 2 | 3 | 4 | 5 | 6 | 7 | 8 | 9 | 10 |
| --- | --- | --- | --- | --- | --- | --- | --- | --- | --- | --- |

الم لا يحتمل لا يوجد ألم

Fig.7

**Assessor Chart**

**Patient No.:**

| Examiner Assignment | Score | | Date | | Follow up by hours and days | |
| --- | --- | --- | --- | --- | --- | --- |
|  |  | |  | | 6 | |
|  |  | |  | | 12 | |
|  |  | |  | | 24 | |
|  |  | |  | | 48 | |
|  |  |  | | 7 days | |  |

Fig. 8

**Patient No.:**

| Examiner Assignment | HU | Date | Follow up by months |
| --- | --- | --- | --- |
|  |  |  | 3 |
|  |  |  | 6 |

Fig. 9

**Patient No.:**

| Examiner Assignment | CFU | | Date | | Readings | |
| --- | --- | --- | --- | --- | --- | --- |
|  |  | |  | | Initial | |
|  |  | |  | | After endodontic treatment | |
|  |  | |  | | After laser irradiation | |
|  |  | |  | | recolonization | |
|  |  |  | | After second endodontic treatment | |  |
|  |  |  | | After second laser irradiation | |  |

Fig. 10

**ألم ما بعد الجراحة و التأثير المضاد للجراثيم و معافاة آفة حوائط الذروة المزمنةالمعالجة بالديود ليزر مقابل الجراحة اللبية**

**(تجربة إكلينيكية بالانتقاء العشوائي)**

مشروع بحث مقدم الى كلية طب الفم والأسنان جامعة القاهرة كجزء متمم من مقومات الحصول على درجة الدكتوراة فى علاج الجذور

مقدم من :

دينا أحمد علي مرسي

ماجستير طب الفم والاسنان

جامعة القاهرة

2013

كلية طب الفم والأسنان

جامعة القاهرة

2015

1. Ultracaine- dental forte, germany [↑](#footnote-ref-1)
2. Maillefer inst, Switzerland [↑](#footnote-ref-2)
3. Dentsply maillefer, USA [↑](#footnote-ref-3)
4. Dentsply maillefer, USA [↑](#footnote-ref-4)
5. MD chelcream, META BIOMED CO., LTD, Korea [↑](#footnote-ref-5)
6. Lite medics, Italy [↑](#footnote-ref-6)
7. -Ultradent, South Jordan, UT, USA [↑](#footnote-ref-7)
8. -Dentsply, Latin America [↑](#footnote-ref-8)
9. dentsply, Maillefer, USA [↑](#footnote-ref-9)
10. 3 M ESPE, St Paul M [↑](#footnote-ref-10)
11. - Septodont, Saint Maur des fosses, Cedex France [↑](#footnote-ref-11)
12. Satellec action group france [↑](#footnote-ref-12)
13. MTA, Angelus, Londrina, Parona, Brazil [↑](#footnote-ref-13)
