# Supplementary material for: Postoperative pain and antibacterial effect of 980 nm diode laser versus conventional endodontic treatment in necrotic teeth with chronic periapical lesions: A randomized control trial
Source: F1000Res. 2018 Nov 15;7:1795. [Version 1] doi: 10.12688/f1000research.16794.1 (PMC6659763; doi:10.12688/f1000research.16794.1)
Supplement: Peer review history

# Peer review: Reviewer response for version 1

**DOI:** 10.5256/f1000research.18360.r49690

**Published:** 2019-07-25

**Authors:** Ibrahim Ethem Yaylali

**Affiliations:** 1 Department of Dentistry, Isparta Military Hospital, Isparta, Turkey

**Introduction:**

 There is a lack of information about the previously performed studies. The authors should clearly and briefly describe the existing studies, which have been done so far. Why did the authors perform this study? Which gaps was/were exist in the field of postoperative pain evaluation when diode lasers are used? What was the Null hypothesis? All should be described in the Introduction. The power analysis is adequate.

**Methods:**

 Allocation concealment and sequence generation were described adequately. 

**Results:**

 Demographic and baseline characteristics (mean age, sex, education, etc) of the patients can be showed in one Table so that Table 1 and Table 2 can be removed.

**Discussion:**

 In the Discussion, the second paragraph is unnecessary. It can be completely removed and can be add to the Introduction.

I confirm that I have read this submission and believe that I have an appropriate level of expertise to confirm that it is of an acceptable scientific standard.

# Peer review: Reviewer response for version 1

**DOI:** 10.5256/f1000research.18360.r49584

**Published:** 2019-07-04

**Authors:** Patricia da Ana

**Affiliations:** 1 Center for Engineering, Modeling and Applied Social Sciences, Federal University of ABC, Sao Bernardo do Campo, Brazil

This is a well-written and well-conducted article with clear problem and objective. For indexing, it is suggested some changes, described below:

1. There are many recent published papers that report the clinical efficacy of diode laser in endodontic treatment (see Genc Sen and, Kaya 2019 ^1^). In this way, the authors need to make clear in the introduction what is the innovation that this manuscript presents.
2. References need to be more up-to-date. There is no citation of any article published in the last 5 years.
3. The authors should pay attention to the citations. In the last sentence of the introduction, they comment that the use of the diode laser in endodontics was recently established, but they cite a 2004 reference.
4. Please replace the abbreviation of units using capital letters; for example, ml to mL, watt to Watt.
5. Please give references to the laser protocol used. Are there studies in the literature based on temperature increases generated in the root canal using such parameters? What are the observed values? Comment on their safety for the periodontal ligaments in the discussion.
6. The authors do not explain how they ensured periods of pain assessment.
7. Please correct the neodymium laser nomenclature. The correct one is Nd:YAG.
8. Please explain why there was no bacterial collection in the S3 period of the placebo group. Discuss whether the insertion of the laser fiber may have exerted some mechanical removal of the biofilm.
9. Please correct the captions of all the figures and tables. There are legends that mention letters and asterisks, but there is nothing in the figure (e.g., Figure 2), while Tables 4 and 5 do not explain what upper and lower case letters refer to.

I confirm that I have read this submission and believe that I have an appropriate level of expertise to confirm that it is of an acceptable scientific standard, however I have significant reservations, as outlined above.

## References

[1] : Effect of Root Canal Disinfection with a Diode Laser on Postoperative Pain After Endodontic Retreatment. *Photobiomodul Photomed Laser Surg*.2019;37(2) : 10.1089/photob.2018.4539 85-90 10.1089/photob.2018.4539 31050927

# Peer review: Reviewer response for version 1

**DOI:** 10.5256/f1000research.18360.r40677

**Published:** 2018-12-03

**Authors:** Eman H.A. Aboulezz

**Affiliations:** 1 Basic Dental Science Department, Oro-dental Research Division, National Research Centre, Cairo, Egypt

After reviewing the manuscript entitled 'Posteroperative pain and antibacterial effect of 980nm diode laser versus conventional nedodontic treatment in necrotic teeth with chronic periapical lesions:A randomized control trial', I have found that it is such an outstanding article overall. It provides a comprehensive examination of a complex subject; providing new options in treatment of such an inevitable problem. A detailed description of the manuscript will be as follows:

**Introduction:** which was a well-written and covering more than enough of the relevant theoretical data of literature.

**Aim of the study:** Here the author stated the objectives and goals of the study in an appropriate manner.

**In the Methods**: the author gave a detailed description of the employed methodology of the investigation. It was very well elaborated and relevant to the aim of the study.

**Results:** The results were well organized into different categories; this section included tables and charts. All of which help the reader get a clear detailed insight of the different aspects occurring within the two groups of patients.

**Discussion: **the author in this chapter was successful in showing logical overall arguments and used appropriate number of bibliography sources. This shows that the author developed a deep understanding of the theoretical knowledge and the discussed problems.

**Conclusion**: The manuscript provides a relevant summary of the subject, methodology and results.

**References**: They were organized and included a lot of recent publications.

 Generally, this manuscript meets all the basic requirements of good research and demonstrates coherence between theoretical position, problem presented and the methodology appointed. The author showed in this manuscript a significant level of understanding and analytical ability. I find the work innovative and recommend indexing it.

I confirm that I have read this submission and believe that I have an appropriate level of expertise to confirm that it is of an acceptable scientific standard.
